# Supplementary material for: Mitigating a TDP-43 proteinopathy by targeting ataxin-2 using RNA-targeting CRISPR effector proteins
Source: Nat Commun. 2023 Oct 14;14:6492. doi: 10.1038/s41467-023-42147-z (PMC10576788; doi:10.1038/s41467-023-42147-z)
Supplement: Supplementary file 1 — Supplementary Information [file 41467_2023_42147_MOESM1_ESM.pdf]

Supplementary Information

Mitigating a TDP-43 proteinopathy by targeting ataxin-2 using RNA-targeting CRISPR effector proteins

M. Alejandra Zeballos C.[1], Hayden J. Moore[1], Jackson E. Powell[1], Tyler J. Smith[1], Najah S. Ahsan[1], Sijia Zhang[1], and Thomas Gaj[1,2,*]

[1] Department of Bioengineering, University of Illinois Urbana-Champaign, Urbana, IL 61801, USA

[2] Carl R. Woese Institute for Genomic Biology, University of Illinois Urbana-Champaign, Urbana, IL 61801, USA

[*] Corresponding author. Email: gaj@illinois.edu

Targeted sequence

crRNA 1: 5'-CTCTCCGTGCATAACTGGAGTCTGTATCTT-3'
crRNA 2: 5'-TTGCGCTGAGAGCAGAGTCAGTAAAAGCAT-3'
crRNA 3: 5'-CTAGTGTTGGGGCCATGCCCCTCCCGGTCA-3'
crRNA 4: 5'-ATATATTTATTGTCCCTAGTGTTGGGGCCA-3'
crRNA 5: 5'-CTCCATTAACTACTCTTTGGTCTGAGCCAG-3'
crRNA 6: 5'-ATGGCCAGGGAACACCTCCATTAACTACTC-3'
crRNA 7: 5'-CTTCTGAAGACATGCGTTTAGGCATAGTAG-3'
crRNA 8: 5'-CCCCACTGACCACTGAGGACCACGTTCCCC-3'
crRNA 9: 5'-CCTCAATAGATGGGGTCAGTGCTCTGTTGG-3'
crRNA 10: 5'-CATAGATTCAGATGTAGAGCTTGGCTGTAA-3'

Supplementary Fig. 1. Sequences of the crRNAs evaluated in this study for their ability to target mATXN2.

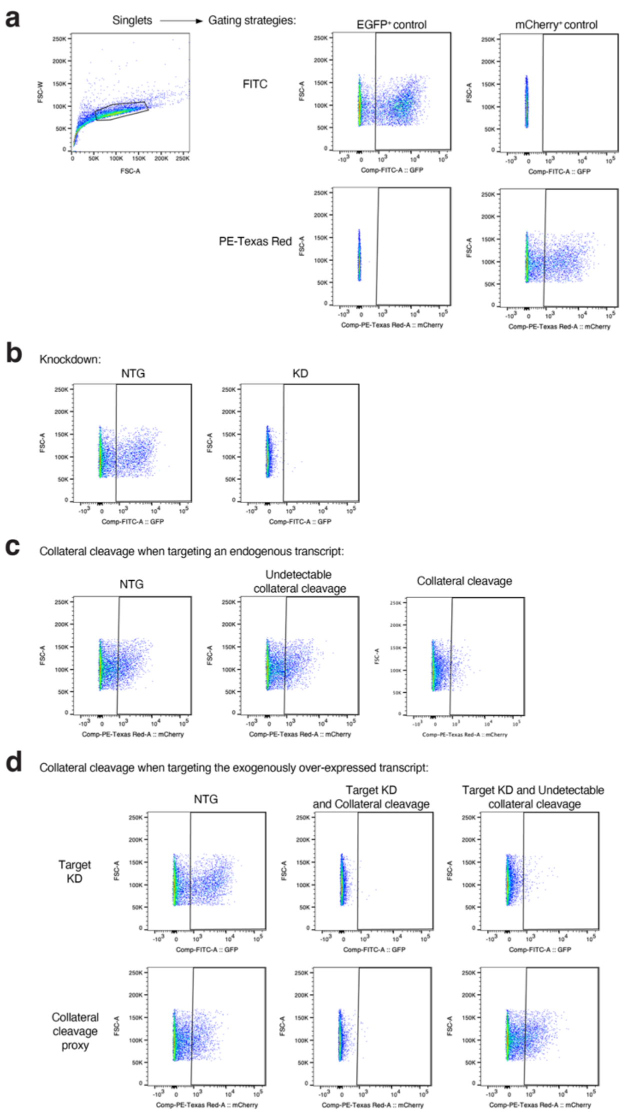

Supplementary Fig. 2. Overview of the gating strategies for the flow cytometry analyses conducted in this study. (a) Representative gates from a flow cytometry analysis. The first gate was used to identify singlets and to exclude dead cells and larger sized objects, while a second fluorescence-based gate was used to identify EGFP- or mCherry-expressing cells. The mean fluorescence intensity (MFI) from EGFP$^+$ and mCherry$^+$ cells was determined using the FITC and PE-Texas Red channels, respectively. **(b-d)** Representative flow cytometry plots used to quantify: **(b)** target knockdown (KD) of a transcript whose expression was linked to EGFP; **(c)** collateral cleavage via mCherry fluorescence; **(d)** collateral cleavage, via mCherry fluorescence, following the target KD of a transcript whose expression was linked to EGFP. Each analysis was compared to a non-targeted (NTG) control.

a

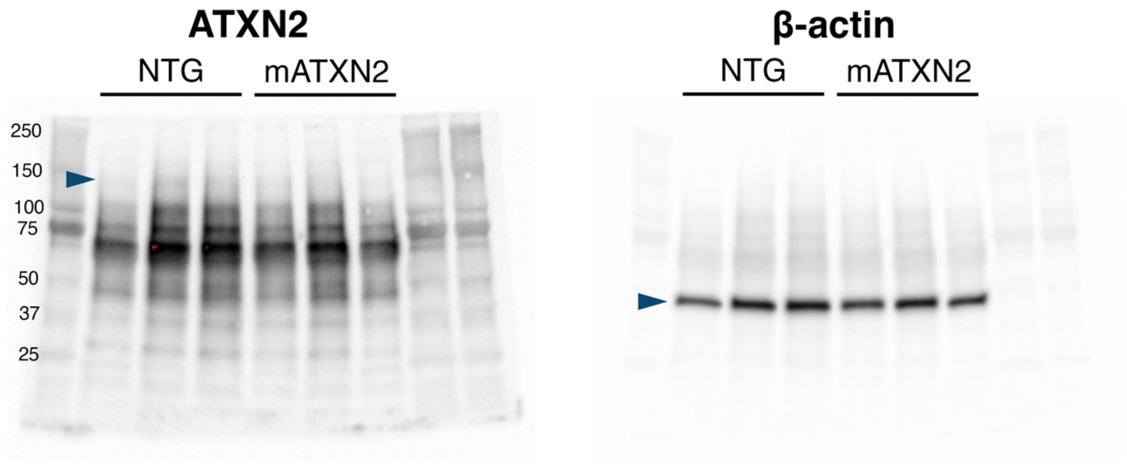

b

ID	ATXN2 Adj. Vol. (Int)	β-actin Adj. Vol. (Int)	Normalized to β-actin	Avg	%	Avg
NTG-1	324,277.02	5,195,651.00	0.062413	0.092466	67.49856	100
NTG-2	918,444.81	8,029,691.00	0.114381		123.7008	
NTG-3	768,384.00	7,637,746.47	0.100603		108.8006	
mATXN2-1	296,795.60	5,465,604.00	0.054302	0.054152	58.72697	58.56381
mATXN2-2	404,264.39	6,497,332.00	0.06222		67.28972	
mATXN2-3	238,326.73	5,188,663.43	0.045932		49.67474	

Supplementary Fig. 3. RfxCas13d can reduce the ataxin-2 protein in Neuro2A cells. (a) Full-membrane probed for (left) the mouse ataxin-2 protein and the (right) β-actin from Neuro2A cells transfected with RfxCas13d alongside either the mATXN2-targeting crRNA-10 or a non-targeted (NTG) crRNA. Neuro2A cells were harvested 48 hr after transfection. Numbers on the left indicate the molecular weight(s) of the components in the ladder, which was loaded in the first lane and the final two lanes, while the blue arrows indicate the target bands. **(b)** Band intensity quantification from Image Lab Software (Bio-Rad). Intensity values for mouse ataxin-2 were normalized to the reference protein in each lane and then normalized to values in cells transfected with RfxCas13d and a NTG crRNA (n = 3). All data points are biologically independent samples.

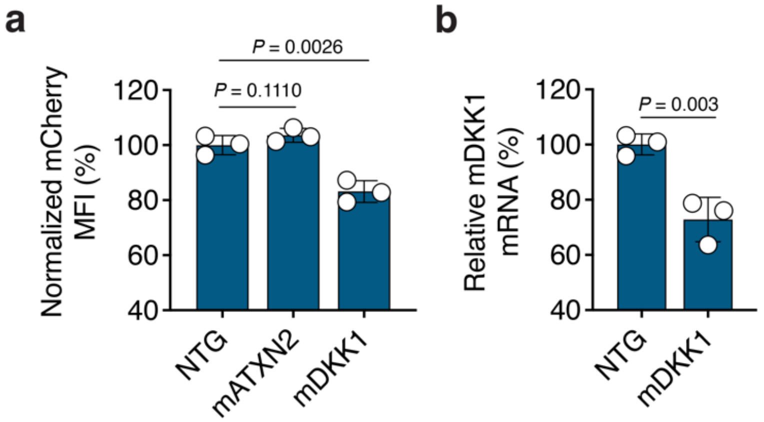

Supplementary Fig. 4. RfxCas13d does not induce collateral cleavage when programmed to target the endogenous mATXN2 transcript in Neuro2A cells. (**a**) Mean fluorescence intensity (MFI) of mCherry in Neuro2A cells transfected with pCAG-mCherry and RfxCas13d alongside either a mATXN2-targeting crRNA or a crRNA targeting the Dickkopf WNT signaling pathway inhibitor 1 (mDKK1) transcript, which served as a positive control for collateral cleavage. Data are normalized to cells transfected with pCAG-mCherry with RfxCas13d alongside a non-targeting (NTG) crRNA (n = 3). (**b**) Relative mDKK1 mRNA from Neuro2A cells transfected with RfxCas13d and crRNA-mDKK1. Data are normalized to cells transfected with RfxCas13d and a NTG crRNA (n = 3). This analysis confirmed that RfxCas13d decreased mDKK1 mRNA. All measurements were conducted at 48 hr post-transfection. Values represent means and error bars indicate SD. Data were compared using a one-tailed unpaired *t*-test, with the exact *P*-values shown. All data points are biologically independent samples.

a

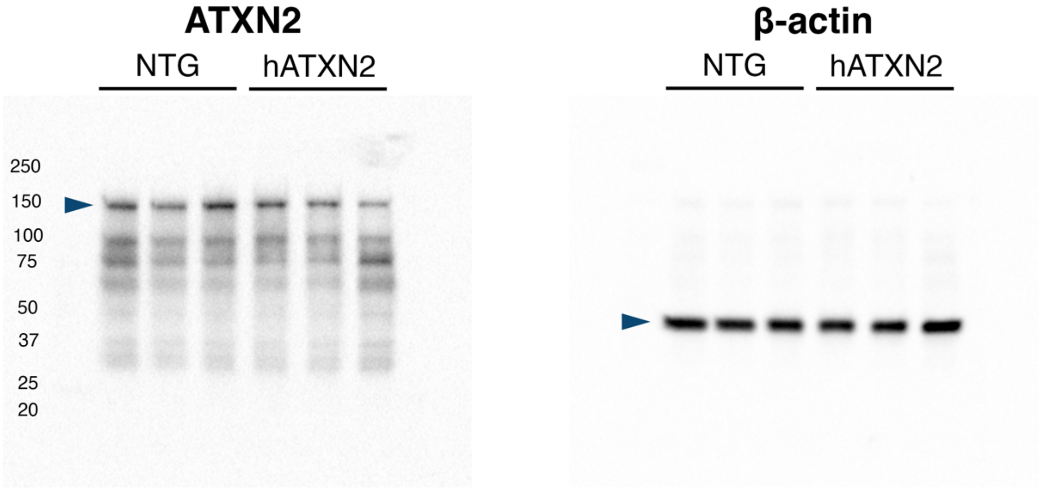

b

ID	ATXN2 Adj. Vol. (Int)	β-actin Adj. Vol. (Int)	Normalized to β-actin	Avg	%	Avg
NTG-1	792,624.00	6,037,384.00	0.131286	0.129616	101.2884	100
NTG-2	570,997.33	5,131,537.17	0.11127218		85.84756	
NTG-3	796,776.00	5,446,556.78	0.14628985		112.864	
hATXN2-1	504,232.84	4,678,633.23	0.10777353	0.077507	83.14832	59.79749
hATXN2-2	455,742.56	5,381,202.05	0.08469159		65.34038	
hATXN2-3	272,436.57	6,801,352.20	0.04005624		30.90377	

Supplementary Fig. 5. RfxCas13d can reduce the ataxin-2 protein in HEK293T cells. (a) Full-membrane probed for (left) the human ataxin-2 protein and the (right) β-actin from HEK293T cells transfected with RfxCas13d alongside either the hATXN2-targeting crRNA-6 or a non-targeted (NTG) crRNA. HEK293T cells were harvested 48 hr after transfection. Numbers on the left indicate the molecular weight(s) of the components in the ladder, while the blue arrows indicate the target bands. **(b)** Band intensity quantification from Image Lab Software (Bio-Rad). Intensity values for human ataxin-2 were normalized to the reference protein in each lane and then normalized to values in cells transfected with RfxCas13d and a NTG crRNA (n = 3). All data points are biologically independent samples.

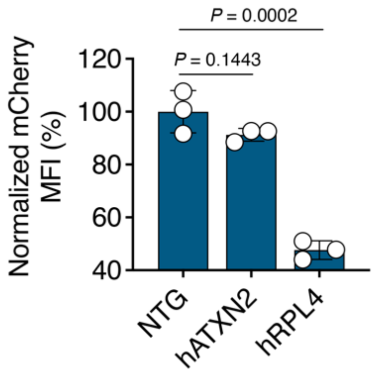

Supplementary Fig. 6. RfxCas13d does not induce collateral cleavage when programmed to target the endogenous hATXN2 transcript in HEK293T cells. (**a**) Mean fluorescence intensity (MFI) of mCherry in HEK293T cells transfected with pCAG-mCherry and RfxCas13d alongside either the hATXN2-targeting crRNA-6 or a validated crRNA targeting RLP4 (ribosomal protein L4)[1], a strongly expressed transcript used here as a positive control for collateral cleavage. Data are normalized to cells transfected with pCAG-mCherry and RfxCas13d with a non-targeted (NTG) crRNA (n = 3). All measurements were conducted at 48 hr post-transfection. Values represent means and error bars indicate SD. Data were compared using a one-tailed unpaired *t*-test, with the exact *P*-values shown. All data points are biologically independent samples.

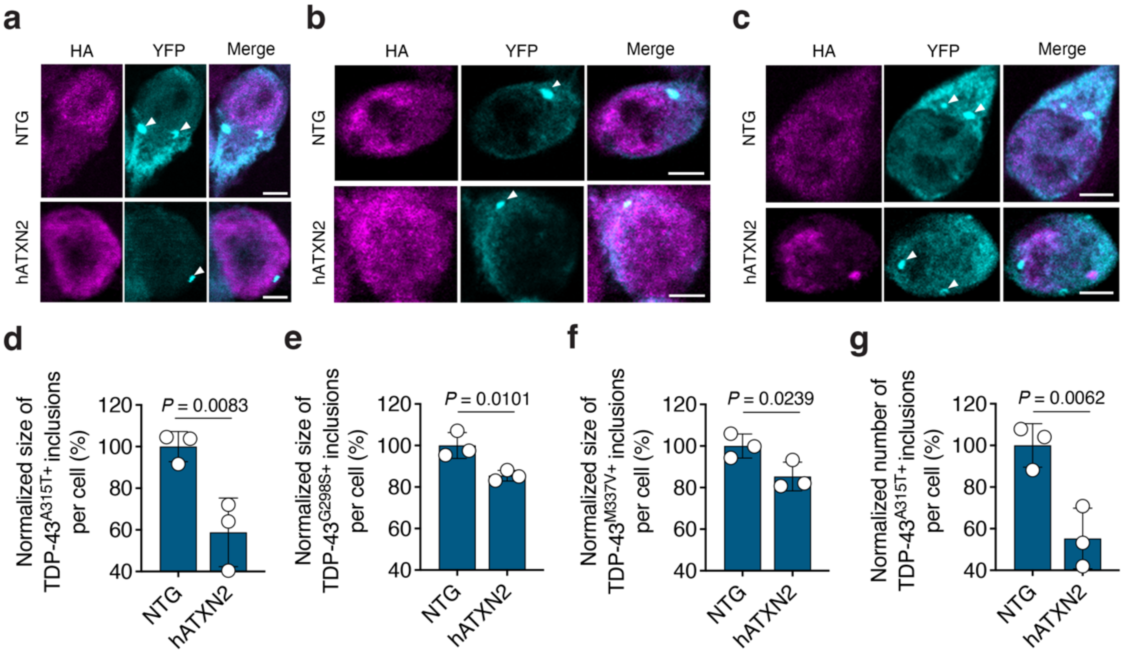

Supplementary Fig. 7. Targeting ATXN2 with RfxCas13d can decrease the aggregation of mutant TDP-43 protein variants. (a-c) Representative immunofluorescent staining of HEK293T cells after transfection with RfxCas13d and the hATXN2-targeting crRNA-6 alongside (**a**) YFP-TDP-43^{A315T}, (**b**) YFP-TDP-43^{G298S}, or (**c**) YFP-TDP-43^{M337V}. Scale bar, 5 µm. White arrowheads indicate inclusions. **(d-g)** Quantification by CellProfiler of the area occupied by (**d**) TDP-43^{A315T+}, (**e**) TDP-43^{G298S+}, and (**f**) TDP-43^{M337V+} foci; and (**g**) the number of TDP-43^{A315T+} foci divided by the number of HA^+ cells analyzed per image. **(d-g)** Data are normalized to cells transfected with YFP-TDP-43 alongside RfxCas13d and a non-targeted (NTG) crRNA (n = 3). All data analyzed at 48 hr post-transfection. >85 cells analyzed per replicate. Values represent means and error bars indicate SD. Data were compared using a one-tailed unpaired *t*-test, with the exact *P*-values shown. All data points are biologically independent samples.

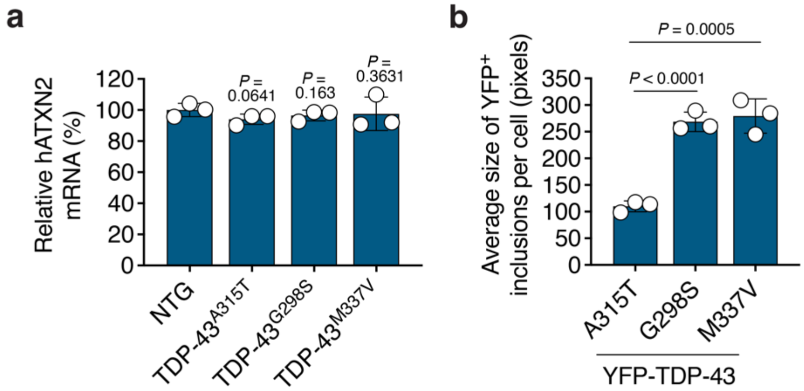

Supplementary Fig. 8. TDP-43 mutants do not affect the relative abundance of hATXN2 and can display varying inclusion sizes in HEK293T cells. (a) Relative hATXN2 mRNA from HEK293T cells transfected with RfxCas13d and the non-targeting (NTG) crRNA alongside YFP-TDP-43^{A315T}, YFP-TDP-43^{G298S}, or YFP-TDP-43^{M337V}. Data are normalized to cells transfected with RfxCas13d and the non-targeting crRNA (n = 3). **(b)** Quantification by CellProfiler of the area occupied by YFP-TDP-43^{A315T+}, YFP-TDP-43^{G298S+}, and YFP-TDP-43^{M337V+} foci (n = 3). >120 cells analyzed per replicate. All data was measured at 48 hr post-transfection. Values represent means and error bars indicate SD. Data were compared using a one-tailed unpaired *t*-test, with the exact *P*-values shown. All data points are biologically independent samples.

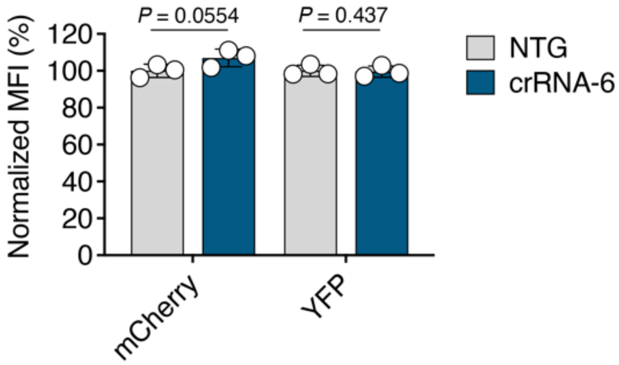

Supplementary Fig. 9. RfxCas13d does not induce collateral cleavage when programmed to target the endogenous hATXN2 in the stress granule (SG) or the ALS-linked TDP-43 variant overexpression assays. Mean fluorescence intensity (MFI) of mCherry and YFP in HEK293T cells transfected with pcDNA3.2-hTDP-43-YFP, pCAG-mCherry and RfxCas13d alongside the hATXN2-targeting crRNA-6. Data are normalized to the MFI from cells transfected with pcDNA3.2-hTDP-43-YFP and pCAG-mCherry with RfxCas13d and a non-targeted (NTG) crRNA (n = 3). All data was measured at 48 hr post-transfection. Values represent means and error bars indicate SD. Data were compared using a one-tailed unpaired *t*-test, with the exact *P*-values shown. All data points are biologically independent samples.

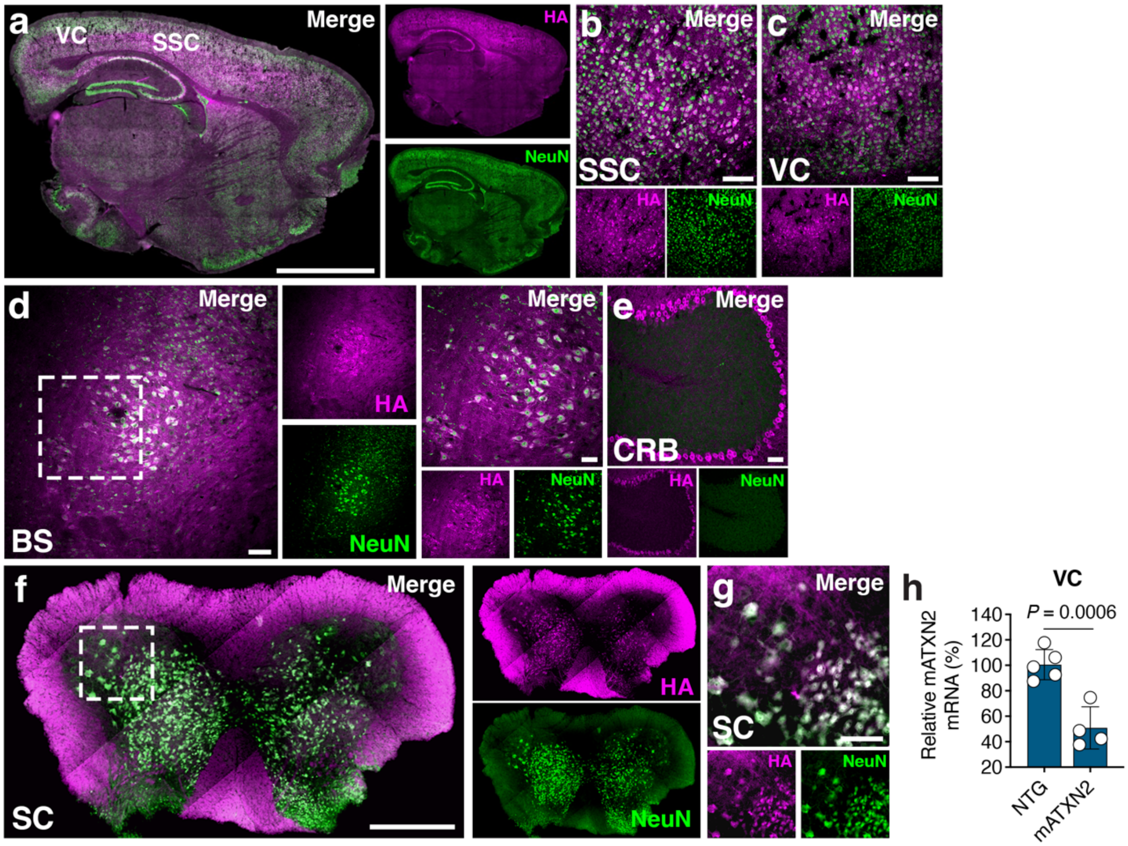

Supplementary Fig. 10. RfxCas13d is expressed in the brain and spinal cord of B6SJLF1/J mice following its AAV delivery. (a-g) Representative immunofluorescence staining of the **(a)** cortex (scale bar, 2 mm), **(b)** the somatosensory cortex (SSC) (scale bar, 50 μm), **(c)** the visual cortex (VC) (scale bar, 50 μm), **(d)** the brain stem (BS) [scale bar, (left) 100 μm, (right) 50 μm], **(e)** the cerebellum (CRB) (scale bar, 50 μm), **(f)** the lumbar spinal cord (SC) (scale bar, 500 μm), and **(g)** the SC anterior horn (scale bar, 100 μm) from B6SJLF1/J mice injected with AAV9-CBh-RfxCas13d-crRNA. **(a, f)** Sections were tile-scanned using an AxioScan.Z1 instrument (Carl R. Woese Institute for Genomic Biology, Univ. Illinois) and stitched together to display a larger view. White boxes denote the adjacent inset. **(h)** Relative mATXN2 mRNA in the VC from B6SJLF1/J mice injected with AAV9-CBh-RfxCas13d-mATXN2. Data are normalized to relative mATXN2 mRNA in the VC from B6SJLF1/J injected with AAV9-CBh-RfxCas13d-NTG. Values represent means and error bars indicate SD (n = 5 for NTG; n = 4 for mATXN2). Data were compared using a one-tailed unpaired *t*-test, with the exact *P*-values shown. All data points are biologically independent samples. Analyses were conducted four weeks post-injection.

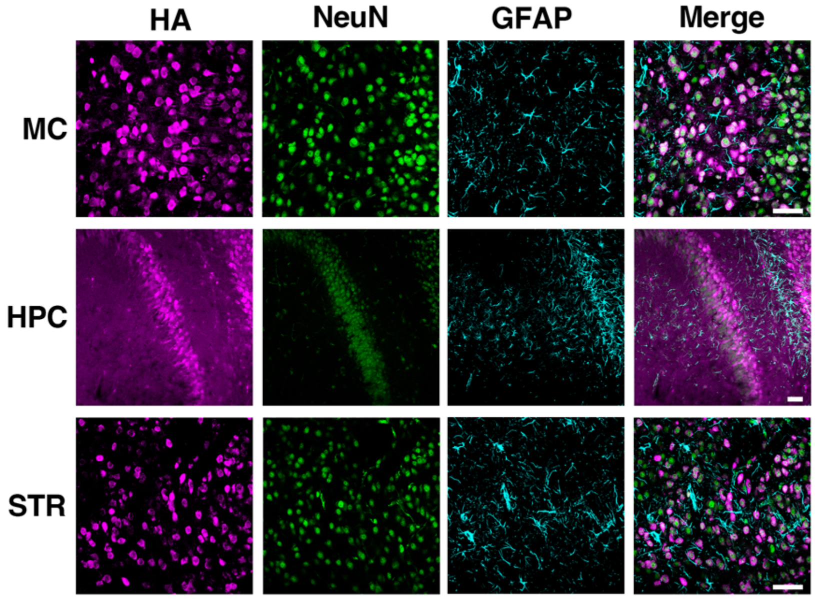

Supplementary Fig. 11. RfxCas13d was expressed in NeuN⁺ cells in B6SJLF1/J mice. Representative immunofluorescence staining of the motor cortex (MC), hippocampus (HPC) and striatum (STR) of B6SJLF1/J mice injected with AAV9-CBh-RfxCas13d-mATXN2 or AAV9-CBh-RfxCas13d-NTG on P1-P2. Scale bar, 50 µm. All analyses were conducted four weeks post-injection.

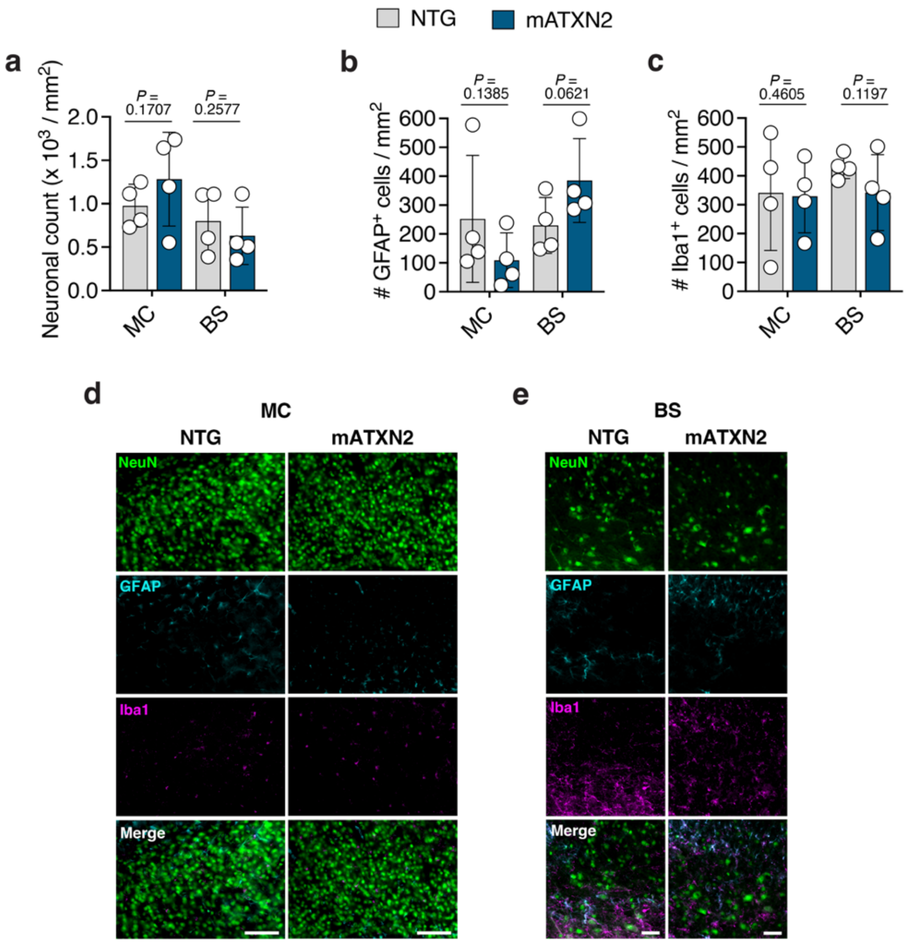

Supplementary Fig. 12. Targeting mATXN2 by RfxCas13d did not affect neuronal viability or trigger an increase in neuroinflammation in the motor cortex or brainstem of B6SJLF1/J mice. Quantification by CellProfiler of immunofluorescence staining for **(a)** NeuN$^+$, **(b)** GFAP$^+$, and **(c)** Iba1$^+$ cells in sections from the motor cortex (MC) and brainstem (BS) of B6SJLF1/J mice injected with AAV9-CBh-RfxCas13d-mATXN2 or AAV9-CBh-RfxCas13d-NTG on P1-P2. Error bars indicate SD (n = 4). For MC, >700 NeuN$^+$ cells, >80 GFAP$^+$ cells, and >500 Iba1$^+$ cells were analyzed per biological replicate. For BS, >304 NeuN$^+$ cells, >178 GFAP$^+$ cells, and >218 Iba1$^+$ cells were analyzed per biological replicate. All analyses were conducted four weeks post-injection. Values represent means and error bars indicate SD. Data were compared using a one-tailed unpaired *t*-test, with the exact *P*-values shown. All data points are biologically independent samples. **(d, e)** Representative immunofluorescence staining of the **(d)** MC and **(e)** BS of B6SJLF1/J mice injected with AAV9-CBh-RfxCas13d-mATXN2 or AAV9-CBh-RfxCas13d-NTG on P1-P2. Scale bar, **(d)** 100 µm and **(e)** 50 µm. Images were captured using identical exposure conditions. All analyses were conducted four weeks post-injection.

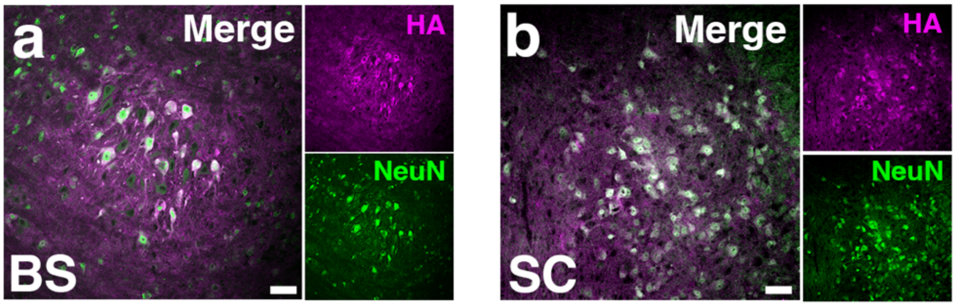

Supplementary Fig. 13. RfxCas13d was detectable in the brain and spinal cord of end-stage TAR4/4 mice. (a, b) Representative immunofluorescence staining of the **(a)** brainstem (BS) and **(b)** spinal cord (SC) from TAR4/4 mice injected with AAV9-CBh-RfxCas13d-crRNA on P1-P2. Scale bar, 50 μm. All analyses were conducted on tissue from animals that progressed to end-stage.

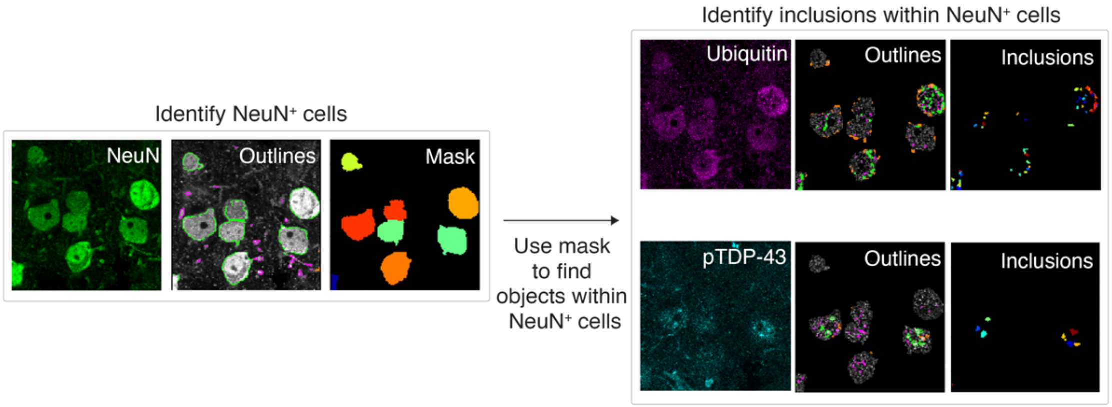

Supplementary Fig. 14. Overview of the CellProfiler pipeline used to identify aggregates in end-stage TAR4/4 mice. Workflow of the automated CellProfiler pipeline used to identify, count, and map cells and/or inclusions in sections from end-stage TAR4/4 mice. The Identify Primary Objects module in CellProfiler was used to create masks for (left) $NeuN^+$ cells. (Right) The Identify Primary Objects module was then used to analyze the $NeuN^+$ cell population for ubiquitin- and pTDP-43-immunoreactive inclusions. Foci ranging from 0.2-2 µm and 0.1-2 µm in sizes were used as the thresholds to identify ubiquitin- and pTDP-43-immunoreactive inclusions respectively.

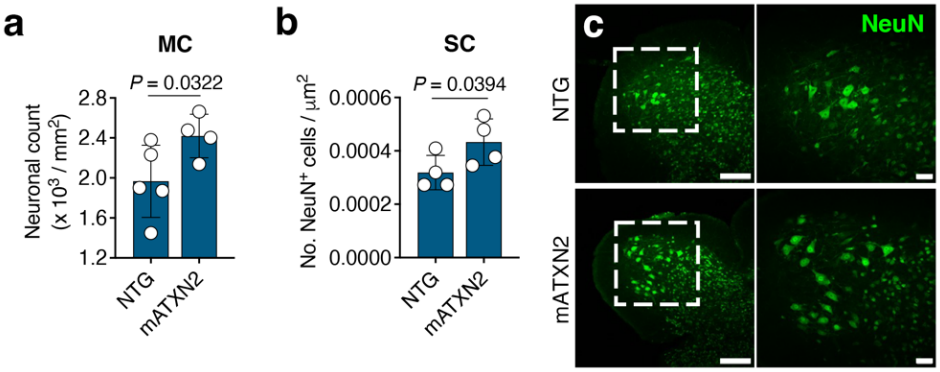

Supplementary Fig. 15. TAR4/4 mice treated with RfxCas13d had an increased number of neurons in the motor cortex and the spinal cord. (a, b) Quantification of immunofluorescence staining of the **(a)** motor cortex (MC) and the **(b)** spinal cord (SC) from TAR4/4 mice injected with AAV9-CBh-RfxCas13d-mATXN2 or AAV9-CBh-RfxCas13d-NTG. Error bars indicate SD (n = 5 for NTG and n = 4 for mATXN2 for MC, n = 4 for SC). Data were compared using a one-tailed unpaired *t*-test, with the exact *P*-values shown. For the MC, >150 cells were analyzed per biological replicate by CellProfiler. For the SC, >105 cells were analyzed per biological replicate (both anterior horns per section and >2 sections per biological replicate). Neuronal viability in the MC was analyzed by CellProfiler, while viability in the SC was determined by a blinded investigator. All data points are biologically independent samples. **(c)** Representative immunofluorescence staining of the SC from TAR4/4 mice injected with AAV9-CBh-RfxCas13d-mATXN2 or AAV9-CBh-RfxCas13d-NTG. Scale bar, (left) 100 µm, (right) 50 µm. All analysis conducted on end-stage tissues from TAR4/4 mice injected at P1-2.

a

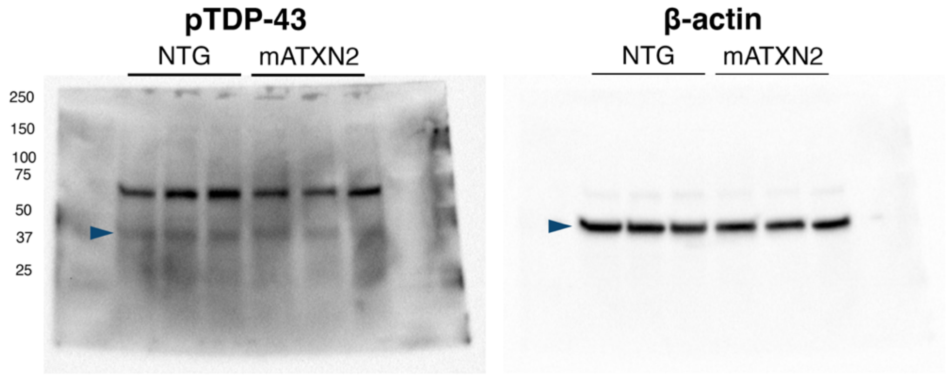

b

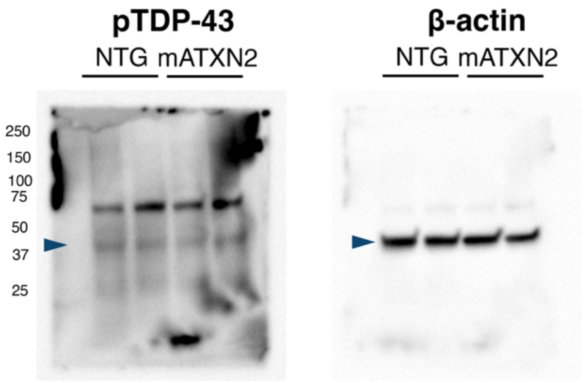

c

ID	pTDP-43 Adj. Vol. (Int)	β-actin Adj. Vol. (Int)	Normalized to β-actin	Avg	%	Avg
NTG-1	1,011,879.43	5,045,495.70	0.200551044	0.302126	66.38001	100
NTG-2	1,132,860.26	4,438,523.77	0.255233568		84.47927	
NTG-3	1,201,130.61	4,208,000.47	0.285439752		94.47716	
NTG-4	1,303,133.21	4,228,223.43	0.308198758		102.0101	
NTG-5	1,446,693.00	3,136,765.82	0.461205294		152.6535	
mATXN2-1	1,110,584.57	5,252,100.62	0.211455311	0.204183	69.98919	67.58226
mATXN2-2	932,563.87	5,234,319.50	0.178163345		58.96994	
mATXN2-3	895,832.73	5,734,101.67	0.156228958		51.70992	
mATXN2-4	891,584.00	3,796,400.54	0.234849824		77.73249	
mATXN2-5	810,398.44	3,373,576.34	0.240219387		79.50975	

Supplementary Fig. 16. RfxCas13d can decrease total pTDP-43 protein in the motor cortex of TAR4/4 mice. (a, b) Full-view of two membranes, present in (**a**) and (**b**), probed for (left) the human pTDP-43 protein and (right) the β-actin from the motor cortex of TAR4/4 mice injected with AAV9-CBh-RfxCas13d-mATXN2. Numbers on the left indicate the molecular weight(s) of the components in the ladder, while the blue arrows indicate the target bands. **(c)** Band intensity quantification from Image Lab Software (Bio-Rad). Intensity values for pTDP-43 were first normalized to the reference protein in each lane and then normalized to values from tissue of TAR4/4 mice injected with AAV9-CBh-RfxCas13d-crRN-NTG (n = 5). All data points are biologically independent samples. All analysis conducted on end-stage tissues from TAR4/4 mice injected at P1-2.

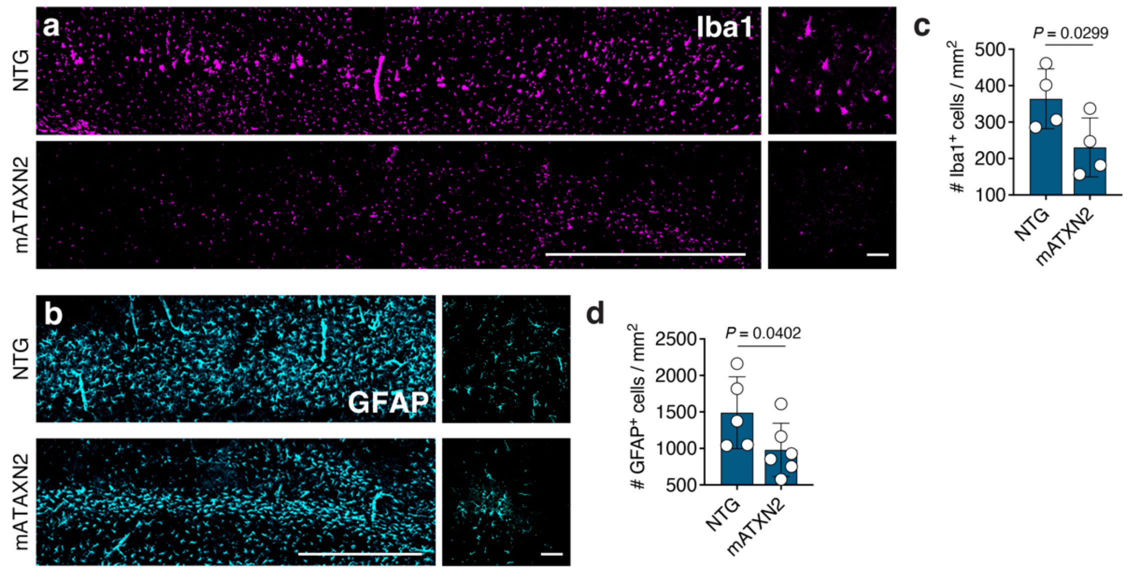

Supplementary Fig. 17. RfxCas13d reduced microgliosis and astrogliosis in TAR4/4 mice. (a and b) Representative immunofluorescence staining of **(a)** Iba1$^+$ cells and **(b)** GFAP$^+$ cells in the motor cortex (MC) from TAR4/4 mice injected with AAV9-CBh-RfxCas13d-mATXN2 and AAV9-CBh-RfxCas13d-NTG. **(c and d)** Quantification by CellProfiler of the immunofluorescence staining of the MC [Iba1 (n = 4); GFAP (n = 5 for NTG and n = 6 for mATXN2]. Values represent means and error bars indicate SD. Data were compared using a one-tailed unpaired *t*-test, with the exact *P*-values shown. All analysis conducted on end-stage tissues from TAR4/4 mice injected at P1-P2. >900 cells were analyzed for the GFAP$^+$ cell quantification per biological replicate. >600 cells were analyzed for the Iba1$^+$ cell quantification per biological replicate. All data points are biologically independent samples.

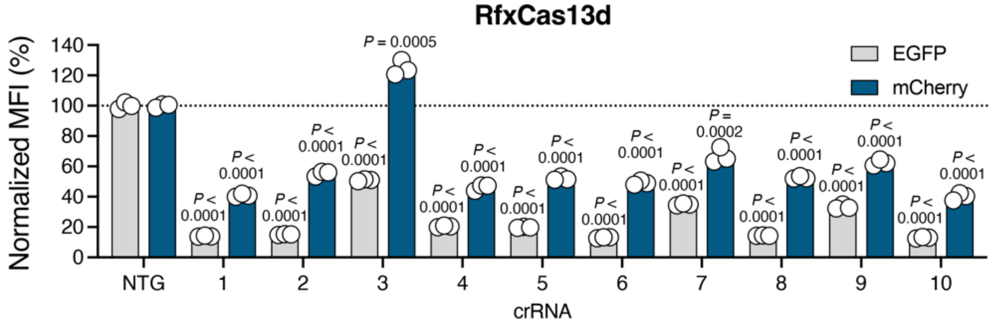

Supplementary Fig. 18. RfxCas13d collaterally *trans*-cleaves mCherry mRNA when programmed to target an overexpressed mRNA in HEK293T cells. EGFP and mCherry mean fluorescence intensity (MFI) in HEK293T cells transfected mATXN2-T2A-EGFP and pCAG-mCherry with an RfxCas13d with mATXN2-targeting crRNAs. Data are normalized to MFI from cells transfected with mATXN2-T2A-EGFP, pCAG-mCherry and RfxCas13d with a non-targeted (NTG) crRNA (n = 3). Values represent means and error bars indicate SD. Data were compared using a one-tailed unpaired *t*-test, with the exact *P*-values shown. All data points are biologically independent samples.

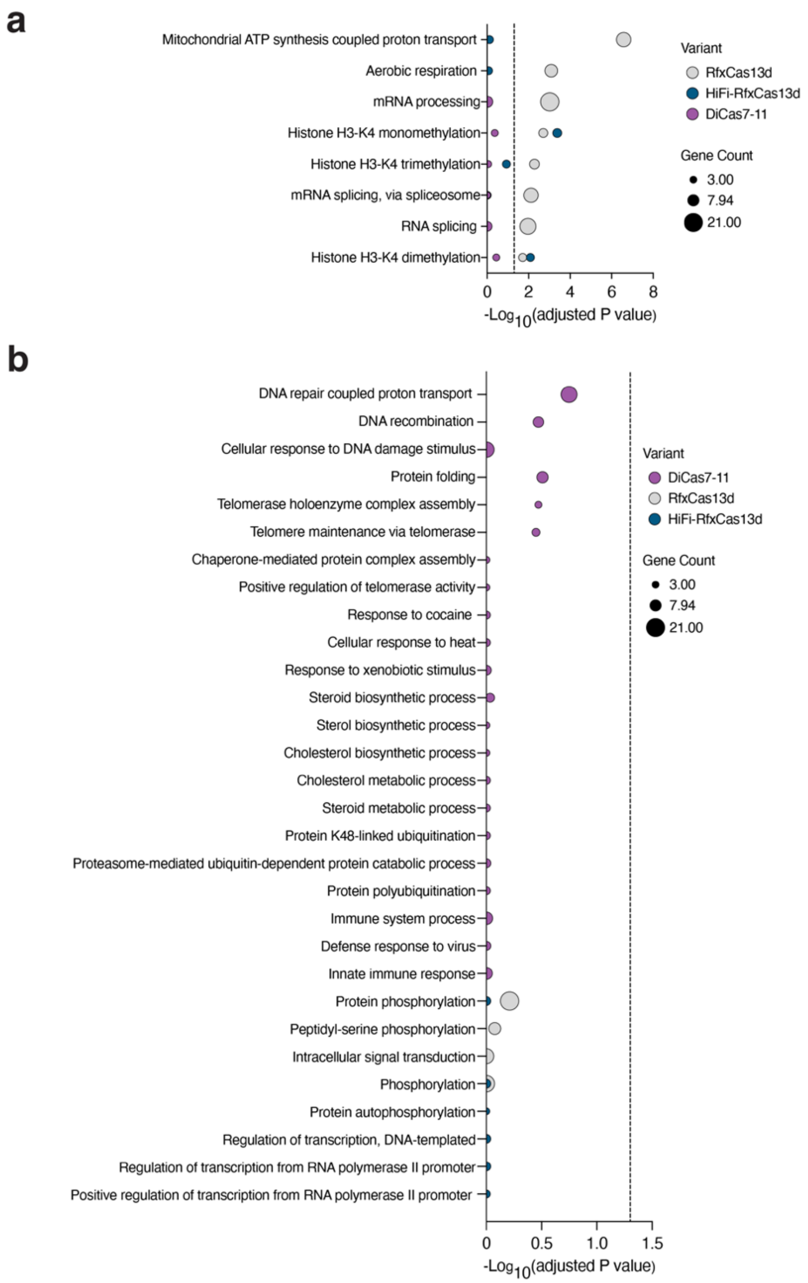

Supplementary Fig. 19. Gene ontology (GO) and biological process (BP) term analysis of the up- and down-regulated DEGs for RfxCas13d, HiFi-RfxCas13d and DiCas7-11. (a-b) Biological process analysis of **(a)** up- and **(b)** down-regulated differentially expressed genes (DEGs) from Neuro2A cells transfected with RfxCas13d, HiFi-RfxCas13d or DiCas7-11 with crRNA-10 relative to cells transfected with each variant and a non-targeted (NTG) crRNA (n = 3). Dotted line denotes the cut-off for values with an FDR-adjusted $P < 0.05$. The list of DEGs for each function are available in **Supplementary Data 3 and Supplementary Data 4**.

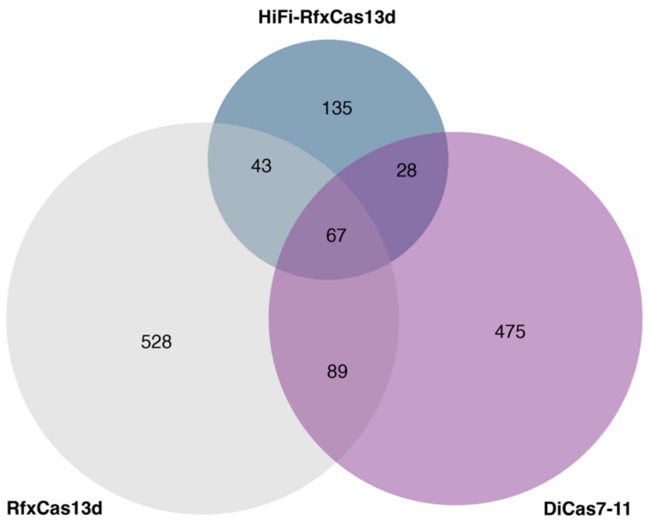

Supplementary Fig. 20. Number of overlapping DEGs between RfxCas13d, HiFi-RfxCas13d and DiCas7-11. Venn diagram of the shared differentially expressed genes (DEGs) from the RNA-seq analysis of Neuro2A cells transfected with RfxCas13d, HiFi-RfxCas13d or DiCas7-11 with the mATXN2-targeting crRNA-10 relative to cells transfected with each variant and a non-targeted (NTG) crRNA (n = 3). The list of DEGs overlapping between RfxCas13d, HiFi-RfxCas13d and DiCas7-11 are available in **Supplementary Data 5**.

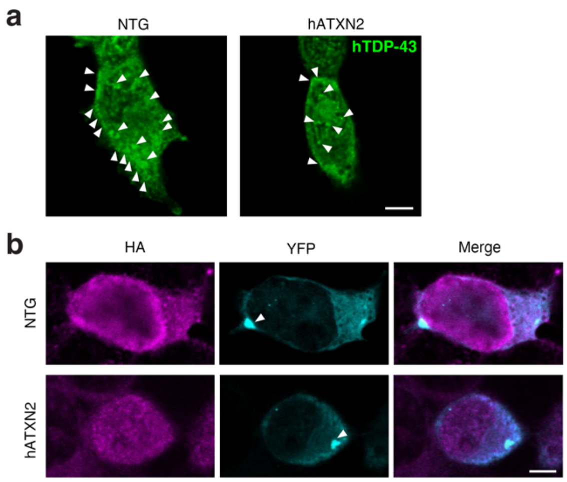

Supplementary Fig. 21. HiFi-RfxCas13d can decrease TDP-43 aggregation. (a) Representative immunofluorescent staining of HEK293T cells 48 hr after transfection with HiFi-RfxCas13d and the hATXN2-targeting crRNA-6 and treatment with sorbitol for 1 hr. >134 cells analyzed per replicate. **(b)** Representative immunofluorescent staining of HEK293T cells 48 hr after transfection with HiFi-RfxCas13d, the hATXN2-targeting crRNA-6 and YFP-TDP-43^{A315T}. >81 cells analyzed per biological replicate by CellProfiler. Images were captured using identical exposure conditions. White arrowheads indicate inclusions. Scale bar, 5 µm.

Supplementary Table 1. Primer sequences used in this study.

Name	Sequence	Utilized for:
NTG	5’-TCACCAGAAGCGTACCATACTC-3’	Fig. 1-4
T2A-GFP Gibson-F	5'-GCAGAAACTCATCTCAGAAGAGGATCTGGCAGCAAATGATATCCTGGATTACAAGGATGACGACGATAAGGTTTCGGGTTCTGGCTCGGAGGGCAGAGGAAGTCTGCTAACATGCGGTGACGTCGAGGAGAATCCTGG-3'	Reporter plasmid mATXN2-T2A-EGFP
T2A-GFP Gibson-R	5'-GGGATGCCACCCGGGATCTGTTCAGGAAACAGCTATGACCGCGGCCGGCCGTTTTTACTTGTACAGCTCGTCCATGCCGAGAGTGATCCCGGCGGCGGTCACGA-3'	Reporter plasmid mATXN2-T2A-EGFP
mGAPDH qPCR-F	5'-AGG TCG GTG TGA ACG GAT TTG-3'	Mouse GAPDH qPCR
mGAPDH-qPCR-R	5'-TGT AGA CCA TGT AGT TGA GGT CA-3'	Mouse GAPDH qPCR
qPCR_mATXN2-F	5’-GCTCCAGGTCCTTCTCCTTGTGC-3’	Mouse ATXN2 qPCR
qPCR_mATXN2-R	5’-AAGATACAGACTCCAGTTATGCACGG- 3’	Mouse ATXN2 qPCR
Mouse mDDK1 crRNA	5’-AGAGAAACAAGGCAATGTAGCACACCTCTA- 3’	Mouse DKK1 targeting
mDKK1-qPCR_F	5'-ATCTGTCTGGCTTGCCGAAAGC-3'	Mouse DKK1 qPCR
mDKK1-qPCR_R	5'-GAGGAAAATGGCTGTGGTCAGAG-3'	Mouse DKK1 qPCR
Human RPL4 Cas13d gRNA-g3	5’-GAAGTTCAGGAACTTCCTCAATACGATGAC- 3’	Human RPL4 targeting
Common_Genotyping	5’- TGA AAT CCG GGT GGT ATT GG-3’	Genotyping of TAR4/4 mice
WT__Genotyping	5’- GGT GAG TTT AAC CTT CAA GGG CT-3’	Genotyping of TAR4/4 mice
Transgene__Genotyping	5’- AGC TTG CTA GCG GAT CCA GAC-3’	Genotyping of TAR4/4 mice
NTG	5’-AAACGGGTCTTCGAGAAGACCT-3’	Fig. 5a, c-i for RfxCas13d variants
NTG	5’-GAACCGAGACCGTGAGGTCTCG-3’	Fig. 5 b,c-e for DiCas7-11
Fusion_NcoI_Forward_v2	5’-CTATTACCATGGTCGAGGTGAGCCCCACGTTCTGCTTCACTC-3’	pAAV-CAG-RfxCas13d-N1V7-U6-crRNA, pAAV-CAG-RfxCas13d-N2V7-U6-crRNA, and pAAV-

		CAG-RfxCas13d-N2V8-U6-crRNA
Fusion_N1V7_R	5'-CCATCCACGCTGGCGCCGAAGTAGGCCTTGGCCAGAGTTTCCTTCAGGCCGAGCATATCCTGCTGGACGGGTCCTGTATACAGAGGGTTG-3'	pAAV-CAG-RfxCas13d-N1V7-U6-crRNA
Fusion_N2V7_R	5'-TGACGGCGTAGGCGGCGTTGGTGGCGTAGGCGGCGAGGGCTTTTTCAATGTCCAGGATGTTATGGATCACCTGGATACAAATATTGTCAT-3'	pAAV-CAG-RfxCas13d-N2V7-U6-crRNA
Fusion_N2V8_R	5'-ACCACGTACACCACGTTGGTAATGTATTCCACGAGGATTTTTCAATGTCCAGGATGTTATGGATCACCTGGATACAAATATTGTCATTG-3'	pAAV-CAG-RfxCas13d-N2V8-U6-crRNA
Fusion_N1V7_F	5'-CTGGCCAAGGCCTACTTCGGCGCCAGCGTGGATGGCAATGACAATATTTGTATCCAGGTGATCCATAACATCCTGGACATTGAAAAAATC-3'	pAAV-CAG-RfxCas13d-N1V7-U6-crRNA
Fusion_N2V7_F	5'-GCCGCCTACGCCACCAACGCCGCCTACGCCGTCAACAATGCCTCCGGCCTGGATAAGGACATTATTGGATTCGGCAAGTTCTCCACAGTG-3'	pAAV-CAG-RfxCas13d-N2V7-U6-crRNA
Fusion_N2V8_F	5'-TCGTGGAATACATTACCAACGTGGTGTACGTGGTCAACAATATCTCCGGCCTGGATAAGGACATTATTGGATTCGGCAAGTTCTCCACAG-3'	pAAV-CAG-RfxCas13d-N2V8-U6-crRNA
Fusion_SacI_Reverse	5'-AGGGCCTTGAGCTCATCATAGGACAGGTTGGTTCCTAAAATACGGATG-3'	pAAV-CAG-RfxCas13d-N1V7-U6-crRNA, pAAV-CAG-RfxCas13d-N2V7-U6-crRNA, and pAAV-CAG-RfxCas13d-N2V8-U6-crRNA
NheI-U6_F	5' GAGGATTGGGCTAGCGAGGGCCTATTTCCCATGATTCCTTCATATTTGCA 3'	pAAV-CAG-DiCas7-11-U6-crRNA
KpnI-polyT7-11_R	5' GGCCGCGGTACCAAAAAAACGAGACCTCACGGTCTCGGTTCCGTGACATCA 3'	pAAV-CAG-DiCas7-11-U6-crRNA
F_DiCas7-11	5'-ATAAAAAGCGAAGCGCGCGGCGGGCGACCGGTGCCACCATGACGACTACTATGAAGATTT-3'	pAAV-CAG-DiCas7-11-U6-crRNA
R_DiCas7-11	5'-AACATCGTATGGGTACGTACGTGCCCACGGTGTCCACGGCGTGGTCAGCTTCTTCTGCCG-3'	pAAV-CAG-DiCas7-11-U6-crRNA
F_BsiWI_3HA	5'-TGGGCACGTACGTACCCATACGATGTTCCAGATTACGCTTACCCTTATGACGTACC-3'	pAAV-CAG-DiCas7-11-U6-crRNA
R_3HA	5'-CAGATGGCTGGCAACTAGAAGGCACAGTCGAAGCTTTCAGGCGTAGTCCGGTACG-3'	pAAV-CAG-DiCas7-11-U6-crRNA

hGAPDH qPCR-F	5’-CCTGACCTGCCGTCTAGAAAA-3’	Human GAPDH qPCR
hGAPDH qPCR-R	5’-CTCCGACGCCTGCTTCAC-3’	Human GAPDH qPCR
qPCR-hAtxn2-E12-F	5’-TAGTCCTGCATCGAACAGAGCTG-3’	Human ATXN2 qPCR
qPCR-hAtxn2-E13-R	5’-AGCTAGGTGATGTTTCATTGGG-3’	Human ATXN2 qPCR

REFERENCES

1. Tong, H. *et al.* High-fidelity Cas13 variants for targeted RNA degradation with minimal collateral effects. *Nat Biotechnol* (2022) doi:10.1038/s41587-022-01419-7.
