# Supplementary material for: Mitigating a TDP-43 proteinopathy by targeting ataxin-2 using RNA-targeting CRISPR effector proteins
Source: Nat Commun. 2023 Oct 14;14:6492. doi: 10.1038/s41467-023-42147-z (PMC10576788; doi:10.1038/s41467-023-42147-z)
Supplement: Supplementary file 2 — Reporting Summary [file 41467_2023_42147_MOESM2_ESM.pdf]

nature portfolio

Corresponding author(s): Thomas Gaj

Last updated by author(s): Sep 26, 2023

Reporting Summary

Nature Portfolio wishes to improve the reproducibility of the work that we publish. This form provides structure for consistency and transparency in reporting. For further information on Nature Portfolio policies, see our Editorial Policies and the Editorial Policy Checklist.

Statistics

For all statistical analyses, confirm that the following items are present in the figure legend, table legend, main text, or Methods section.

n/a	Confirmed	
☐	☒	The exact sample size (*n*) for each experimental group/condition, given as a discrete number and unit of measurement
☐	☒	A statement on whether measurements were taken from distinct samples or whether the same sample was measured repeatedly
☐	☒	The statistical test(s) used AND whether they are one- or two-sided<br>*Only common tests should be described solely by name; describe more complex techniques in the Methods section.*
☒	☐	A description of all covariates tested
☐	☒	A description of any assumptions or corrections, such as tests of normality and adjustment for multiple comparisons
☐	☒	A full description of the statistical parameters including central tendency (e.g. means) or other basic estimates (e.g. regression coefficient) AND variation (e.g. standard deviation) or associated estimates of uncertainty (e.g. confidence intervals)
☐	☒	For null hypothesis testing, the test statistic (e.g. *F*, *t*, *r*) with confidence intervals, effect sizes, degrees of freedom and *P* value noted<br>*Give P values as exact values whenever suitable.*
☒	☐	For Bayesian analysis, information on the choice of priors and Markov chain Monte Carlo settings
☒	☐	For hierarchical and complex designs, identification of the appropriate level for tests and full reporting of outcomes
☒	☐	Estimates of effect sizes (e.g. Cohen's *d*, Pearson's *r*), indicating how they were calculated

Our web collection on statistics for biologists contains articles on many of the points above.

Software and code

Policy information about availability of computer code

Data collection	BD LSRFortessa Cell Analyzer (Roy J. Carver Biotechnology Center Flow Cytometry Facility, Univ. Illinois)<br>Bio-Rad CFX96 qPCR<br>Leica TCS SP8 confocal microscope and a Zeiss Observer Z1 microscope (both at the Beckman Institute for Advanced Science and Technology, Univ. Illinois)<br>AxioScan.Z1 (Carl R. Woese Institute for Genomic Biology, Univ. Illinois)<br>NovaSeq 6000 for RNA sequencing
Data analysis	FlowJo v10 (FlowJo, LLC)<br>Prism 9 (GraphPad) for graphing and statistical analyses<br>CellProfiler 4.2.1 for inclusion and cell counting<br>bcl2fastq v2.20 Conversion Software (Illumina) to generate and demultiplex FASTQ files<br>FastQC (version 0.11.8) to evaluate quality of demultiplexed FastQ files<br>Salmon3 version 1.5.2 for RNA-seq analysis<br>DAVID for overrepresentation analysis and functional annotation (https://david.ncifcrf.gov/home.jsp)<br>BioVenn to produce the Venn diagrams (https://www.biovenn.nl)

For manuscripts utilizing custom algorithms or software that are central to the research but not yet described in published literature, software must be made available to editors and reviewers. We strongly encourage code deposition in a community repository (e.g. GitHub). See the Nature Portfolio guidelines for submitting code & software for further information.

Data

Policy information about availability of data

All manuscripts must include a data availability statement. This statement should provide the following information, where applicable:

- Accession codes, unique identifiers, or web links for publicly available datasets
- A description of any restrictions on data availability
- For clinical datasets or third party data, please ensure that the statement adheres to our policy

All materials generated from this project will be made freely available with no restrictions. RNA-seq data are provided in the Supplementary Information. Overrepresentation analysis and functional annotation was performed using DAVID (the database for annotation, visualization and integrated discovery) to identify the enriched biological terms associated with the differentially expressed genes. A source data file containing the data and statistical values for all the figures in this study are provided with this paper. The unprocessed fluorescence microscopy images for figures 2, 3 and 4 are available from Figshare at DOI: 10.6084/m9.figshare.24187503, while the unprocessed fluorescence microscopy images for the figures in the Supplementary Information are available from Figshare at DOI: 10.6084/m9.figshare.24195102. All source material and unprocessed images are also available upon request from the corresponding author.

Research involving human participants, their data, or biological material

Policy information about studies with human participants or human data. See also policy information about sex, gender (identity/presentation), and sexual orientation and race, ethnicity and racism.

Reporting on sex and gender	N/A
Reporting on race, ethnicity, or other socially relevant groupings	N/A
Population characteristics	N/A
Recruitment	N/A
Ethics oversight	N/A

Note that full information on the approval of the study protocol must also be provided in the manuscript.

Field-specific reporting

Please select the one below that is the best fit for your research. If you are not sure, read the appropriate sections before making your selection.

[x] Life sciences [ ] Behavioural & social sciences [ ] Ecological, evolutionary & environmental sciences

For a reference copy of the document with all sections, see nature.com/documents/nr-reporting-summary-flat.pdf

Life sciences study design

All studies must disclose on these points even when the disclosure is negative.

Sample size	For all in vitro experiments, three independent biological replicates were used at minimum. No statistical method was used to predetermine sample size for the in vitro experiments.<br>For in vivo experiments, the expected effect and SD were informed from published literature (Becker et al. Nature 544, 367-371, 2017). From this information, the target sample size for behavior measurements was determined by power calculations using a = 0.05 and b = 0.80. The sample size reflects the number of independent biological replicates.
Data exclusions	1. For qPCR, if no or minimal amplification was observed for the housekeeping transcript (GAPDH), the samples were repeated. If no or minimal application of the housekeeping transcript was observed again, the sample was excluded.<br>2. For SG analysis, images that were identified to have less than two cells by Cell Profiler and/or presenting an imaging artifact that posed risk for confounding the analysis, were excluded from the blinded analysis.<br>3. For the quantification of pathological hallmarks in mouse tissues, images with artifacts were excluded from the analysis to avoid Cell Profiler identifying them as cells or inclusions. Only one mouse, which had a severally undersized brain and was in the negative control group, was excluded from the blinded analysis. This animal was replaced by another mouse for this analysis.
Replication	1. All in vitro studies were performed with at least 3 independent biological replicates. 2-3 technical replicates per independent biological replicate were conducted for all qPCR measurements. The number of independent biological replicates is specified in the Figure Legends.<br>2. For all behavior, each session for each animal comprised of 3 independent trials, which were averaged and normalized to the day 16 value for that animal. The number of replicates for each measurement are indicated in the Figure Legends.<br>3. For analyses involving cell counting, >80 cells were counted for each in vitro biological replicate and >80 cells were counted for each in vivo biological replicate.<br>All attempts at replication were successful for cell counting experiments.

Randomization	Mice were randomized by sex into treatment groups. Sex of neonatal mice was not determined before injections. Experiments other than those involving mice were not randomized and the proper statistical analysis was performed as indicated in the "Statistical analysis and reproducibility section."
Blinding	Image acquisition, automated quantification of inclusions and cell counts by Cell Profiler, and by cell counts by observation were performed by a blinded investigator. All behavior measurements and behavior scoring were performed by a blinded investigator.

Reporting for specific materials, systems and methods

We require information from authors about some types of materials, experimental systems and methods used in many studies. Here, indicate whether each material, system or method listed is relevant to your study. If you are not sure if a list item applies to your research, read the appropriate section before selecting a response.

Materials & experimental systems

n/a	Involved in the study	
☐	☒	Antibodies
☐	☒	Eukaryotic cell lines
☒	☐	Palaeontology and archaeology
☐	☒	Animals and other organisms
☒	☐	Clinical data
☒	☐	Dual use research of concern
☒	☐	Plants

Methods

n/a	Involved in the study	
☒	☐	ChIP-seq
☐	☒	Flow cytometry
☒	☐	MRI-based neuroimaging

Antibodies

Antibodies used

In vitro studies: Rabbit anti-G3BPI (1:500; MLB International, RN048PW), mouse anti-TDP-43 (1:100; clone 2E2-D3, Abnova, H00023435-M01), chicken anti-HA (1:Abcam, ab9111), rabbit anti-ubiquitin (1:200, Proteintech, 10201-2-AP), rabbit anti-ataxin-2 polyclonal antibody (1:500; Proteintech, 21776-1-AP) for mouse ataxin-rabbit anti-ataxin-2 polyclonal antibody (1:2000; Novus Biologicals, NBP1-90063) for human ataxin-2; and rabbit anti-beta-actin monoclonal antibody (1:1000; Cell Signaling Technology, 4970L).
Tissue analysis: Rabbit anti-HA (1:500, Cell Signaling Technology, C29P4), chicken anti-GFAP (1:1000, Abeam, ab4674), goat anti-lbal (1:800, NOVUS, NB100-1028SS), mouse anti-NeuN (1:500, EMD Millipore Corp, MAB377), mouse anti-pTDP-43 (1:3000, CosmoBio, ps409/410), and chicken anti-NeuN (1:500, EMD Millipore Corp, ABN91), rabbit anti-pTDP-43 (pS409/410) polyclonal antibody (1:1000; CosmoBio, CAC-TIP-PTD-P07).

Validation

In vitro studies:
- Rabbit anti-G3BPI was used by the following references: Sundararaman B et al. Resources for the Comprehensive Discovery of Functional RNA Elements. Mol Cell. 61, 903-13 (2016) (PMID:Context-Dependent and Disease-Specific Diversity in Protein Interactions within Stress Granules. Cell 172, 590-604.eB (2018) (PMID:29373831); Koyuncu S et al. The ubiquitin ligase UBR5 collapse in pluripotent stem cells from Huntington's disease patients. Nat Commun. 9, 2886 (2018)(PMID:30038412))
- Mouse anti-TDP-43 was used by 11 references on supplier website: https://www.sigmaaldrich.com/US/en/product/sigma/wh0023435ml
- Chicken anti-HA was used by 15 references on supplier website: https://www.abcam.com/products/primary-antibodies/ha-tagantibody-ab9lll.html
- Rabbit anti-ubiquitin was used by 310 references on supplier website: https://www.ptglab.com/products/ubiquitin-Antibody-10201-2-AP.htm
- Rabbit anti-ataxin-2 polyclonal antibody (1:500; Proteintech, 21776-1-AP) was used by 43 references on supplier website: https://www.ptglab.com/products/ATXN2-Antibody-21776-1-AP.
- Rabbit anti-ataxin-2 polyclonal antibody (1:2000; Novus Biologicals, NBP1-90063) for human ataxin-2was used by 6 references on supplier website: https://www.novusbio.com/products/ataxin
- Rabbit anti-beta-actin monoclonal antibody (1:1000; Cell Signaling Technology, 4970L) used by 5636 references on supplier website: https://www.cellsignal.com/products/primary-antibodies/_requestid=3764601
Tissue analysis:
- Rabbit anti-HA was used by 2244 references on supplier website: https://www.cellsignal.com/products/primary-antibodies/ha-tagc29f4-
rabbit-mab/3724
- Chicken anti-GFAP was used by 405 references on supplier website: https://www.abcam.com/products/primary-antibodies/gfapantibody-
ab4674.html
- Goat anti-lbal was used by 274 references on supplier website: https://www.novusbio.com/products/aif-l-ibalantibody_
nbl00-1028
- Mouse anti-NeuN was used by 4601 references on supplier website: https://www.sigmaaldrich.com/US/en/product/mm/mab377
- Mouse anti-pTDP-43 used by lnukai, Y. et al. Abnormal phosphorylation of Ser409/410 of TDP-43 in FTLD-U and ALS. FEBS Lett. 582, 2899-2904 (2008) and provider:
https://www.fishersci.com/shop/products/NC0877946/NC0877946
- Chicken anti-NeuN was used by 101 references on supplier website: https://www.sigmaaldrich.com/US/en/product/mm/abn9l

	- Rabbit anti-pTDP-43 (pS409/410) polyclonal antibody (1:1000; CosmoBio, CAC-TIP-PTD-P07) used by Hasegawa Y, Miyamoto K, Yazaki C & Igarashi M (1981) Endocrinology l09: 130-135 and Kurosumi K (1988) Endocrionology 122: 2803-2808in, supplier website: https://www.cosmobiousa.com/products/anti-phospho-tdp-43-ps409-410-pab

Eukaryotic cell lines

Policy information about cell lines and Sex and Gender in Research

Cell line source(s)	HEK293T cells (American Type Culture Collection) and Neuro2A cells (a gift from Pablo Perez-Pinera)
Authentication	HEK293T were authenticated as they were purchased from ATCC. HEK293T and Neuro2A cells were further authenticated cell morphology and species-specific PCR and qPCR primers.
Mycoplasma contamination	The cell lines were not tested for mycoplasma contamination.
Commonly misidentified lines (See ICLAC register)	No commonly misidentified lines were used in this study.

Animals and other research organisms

Policy information about studies involving animals; ARRIVE guidelines recommended for reporting animal research, and Sex and Gender in Research

Laboratory animals	Wild type mice: B6SJLF1/J; Jackson Laboratory, Stock No. 100012<br>TAR4/4 transgenic mice: B6;SJL-Tg(Thyl-TARDBP)4Singh/J; Jackson Laboratory, Stock No. 012836<br>Mice were housed on a light:dark 12:12-hour cycle and maintained at a temperature range of 18-26 °C.<br>All mice were injected at P1-2 and harvested after 4 weeks or at end-stage.
Wild animals	No wild animals were used in this study
Reporting on sex	Both male and female mice were used in the study. Neonatal mice were randomly allocated to the treatment groups. At weaning, sex was determined by visual differences between female and male mice based on the distance between the external genitalia and the anus.
Field-collected samples	No field-collected samples were used in this study.
Ethics oversight	All animal procedures were approved by the Illinois Institutional Animal Care and Use Committee (IACUC) at the University of Illinois and conducted in accordance with the National Institutes of Health Guide for the Care and Use of Laboratory Animals.

Note that full information on the approval of the study protocol must also be provided in the manuscript.

Flow Cytometry

Plots

Confirm that:

- [x] The axis labels state the marker and fluorochrome used (e.g. CD4-FITC).
- [x] The axis scales are clearly visible. Include numbers along axes only for bottom left plot of group (a 'group' is an analysis of identical markers).
- [x] All plots are contour plots with outliers or pseudocolor plots.
- [x] A numerical value for number of cells or percentage (with statistics) is provided.

Methodology

Sample preparation	After 48 hours post-transfection, cells were detached from wells using trypsin, pelleted in individual epi tubes, washed, resuspended and diluted to 1x10^6 cells/mL in PBS. Each sample was strained into single-cell suspensions using Falcon Round-Bottom Polystyrene Test Tubes with Cell Strainer Snap Caps (Corning) and vortexed before being placed in the cell analyzer instrument.
Instrument	BD LSRFortessa Cell Analyzer (Roy J. Carver Biotechnology Center Flow Cytometry Facility, Univ. Illinois)
Software	FlowJo v10 (FlowJo, LLC)
Cell population abundance	A total of 20,000 events were recorded for each replicate.
Gating strategy	Untreated cells were used as a negative control, cells transfected with EGFP only or mCherry only were used as controls for FITC and PE/Texas Red respectively.

- [x] Tick this box to confirm that a figure exemplifying the gating strategy is provided in the Supplementary Information.
