# Supplementary material for: Mitigating a TDP-43 proteinopathy by targeting ataxin-2 using RNA-targeting CRISPR effector proteins
Source: Nat Commun. 2023 Oct 14;14:6492. doi: 10.1038/s41467-023-42147-z (PMC10576788; doi:10.1038/s41467-023-42147-z)
Supplement: Supplementary file 3 — Description of Additional Supplementary Files [file 41467_2023_42147_MOESM3_ESM.pdf]

Title: Supplementary Data 1.

Description: Processed RNA-seq data.

Title: Supplementary Data 2.

Description: Gene ontology (GO) and biological process (BP) term analysis of DEGs for RfxCas13d, HiFi-RfxCas13d and DiCas7-11.

Title: Supplementary Data 3.

Description: Gene ontology (GO) and biological process (BP) term analysis of up-regulated DEGs for RfxCas13d, HiFi-RfxCas13d and DiCas7-11.

Title: Supplementary Data 4.

Description: Gene ontology (GO) and biological process (BP) term analysis of down-regulated DEGs for RfxCas13d, HiFi-RfxCas13d and DiCas7-11.

Title: Supplementary Data 5.

Description: List of DEGs and overlapping DEGs between RfxCas13d, HiFi-RfxCas13d and DiCas7-11.

Title: Supplementary Data 6.

Description: Gene ontology (GO) and biological process (BP) term analysis of overlapping DEGs between RfxCas13d, HiFi-RfxCas13d and DiCas7-11.
